# HDAC4/5-HMGB1 signalling mediated by NADPH oxidase activity contributes to cerebral ischaemia/reperfusion injury

**DOI:** 10.1111/jcmm.12040

**Published:** 2013-03-11

**Authors:** Min He, Bin Zhang, Xinbing Wei, Ziying Wang, Baoxia Fan, Pengchao Du, Yan Zhang, Wencheng Jian, Lin Chen, Linlin Wang, Hao Fang, Xiang Li, Ping-An Wang, Fan Yi

**Affiliations:** a Department of Pharmacology, Shandong University School of MedicineJinan, China; b Department of Radiology, Qilu Hospital, Shandong UniversityJinan, China; c Department of Medicinal Chemistry, Key Laboratory of Chemical Biology, Ministry of Education, School of Pharmacy, Shandong UniversityJinan, China

**Keywords:** Histone deacetylase, Stroke, HMGB1, Reactive oxygen species

## Abstract

Histone deacetylases (HDACs)-mediated epigenetic mechanisms play critical roles in the homeostasis of histone acetylation and gene transcription. HDAC inhibitors have displayed neuroprotective properties in animal models for various neurological diseases including Alzheimer's disease and ischaemic stroke. However, some studies have also reported that HDAC enzymes exert protective effects in several pathological conditions including ischaemic stress. The mixed results indicate the specific roles of each HDAC protein in different diseased states. However, the subtypes of HDACs associated with ischaemic stroke keep unclear. Therefore, in this study, we used an *in vivo* middle cerebral artery occlusion (MCAO) model and *in vitro* cell cultures by the model of oxygen glucose deprivation to investigate the expression patterns of HDACs and explore the roles of individual HDACs in ischaemic stroke. Our results showed that inhibition of NADPH oxidase activity ameliorated cerebral ischaemia/reperfusion (I/R) injury and among Zn^2+^-dependent HDACs, HDAC4 and HDAC5 were significantly decreased both *in vivo* and *in vitro*, which can be reversed by NADPH oxidase inhibitor apocynin. We further found that both HDAC4 and HDAC5 increased cell viability through inhibition of HMGB1, a central mediator of tissue damage following acute injury, expression and release in PC12 cells. Our results for the first time provide evidence that NADPH oxidase-mediated HDAC4 and HDAC5 expression contributes to cerebral ischaemia injury *via* HMGB1 signalling pathway, suggesting that it is important to elucidate the role of individual HDACs within the brain, and the development of HDAC inhibitors with improved specificity is required to develop effective therapeutic strategies to treat stroke.

## Introduction

Despite considerable studies have demonstrated that multiple pathogenic mechanisms are involved in ischaemic stroke, including inflammation, oxidative stress, excitotoxicity, calcium overload, apoptosis and disruption of the blood–brain barrier 1, the pre-clinical protective agents targeting a specific pathway failed to demonstrate clinical efficacy 2. Therefore, achieving therapeutic efficacy in ischaemic stroke represents one of the biggest challenges in translational neurobiology. Epigenetic mechanisms have been shown to be critical for the regulation of central nervous system (CNS). DNA methylation, histone modifications and chromatin remodelling and non–protein-coding RNAs (ncRNAs) have taken centre stage on orchestrating almost every aspect of cell proliferation, apoptosis and migration, neurogenesis and neural network integration. Thus, the selective modulation of epigenetic processes in stroke may represent a novel but complementary strategy for treatment 3. Among them, histone deacetylases (HDACs) play a key role in the homeostasis of histone acetylation and gene transcription. HDACs are a family of enzymes, which compete for control of the acetylation of lysine residues making up the histones with histone acetyltransferases (HATs), determining the post-translational acetylation status of chromatin and a number of other non-histone proteins 4. To date, at least 18 mammal HDACs have been identified and grouped into four classes 5. Classical HDACs (class I, II and IV) share sequence similarity and are dependent on Zn^2+^ for enzymatic activity; whereas the class III sirtuins (SIRT1-7), which deacetylate many non-histone proteins, act through a distinct NAD+-dependent mechanism. Class I HDACs (HDAC1, 2, 3 and 8) are ubiquitously expressed, mainly localized to the nucleus, and act as transcriptional repressors by deacetylation of chromatin histone and other DNA-binding proteins. Class II HDACs (HDAC4, 5, 6, 7, 9 and 10) are expressed stage and tissue specifically and shuttle between the cytoplasm and the nucleus in response to certain cellular signals, which are divided into two subclasses based on domain organization, IIa (HDAC4, 5, 7 and 9) and IIb (HDAC6 and 10). HDAC11 is the sole member of the class IV HDACs family, which shares sequence similarities with class I and class II HDACs.

There is accumulating evidence that HDAC inhibitors exhibit neuroprotective and neuroregenerative properties in animal models of various neurological diseases including Alzheimer's disease and ischaemic stroke 6, 7. However, recent studies have also provided evidence that HDACs serve very distinct functions within the brain. Reports have indicated that some Zn^2+^-dependent HDACs protect neurons and that inhibition of HDACs activity contributes to the promotion of neuronal death in neurodegenerative disorders rather than beneficial effects 8. It should be noted that currently available HDAC inhibitors are mostly non-selective and inhibit multiple HDAC proteins, and the effects of HDAC inhibitors are often studied by examining changes in bulk histone acetylation or the therapeutic effect observed in a given experimental model 9. Therefore, the use of pan-HDAC inhibitors could be problematic. It is necessary to explore the role of individual HDAC in ischaemic stroke.

A number of studies have demonstrated that NADPH oxidase-derived reactive oxygen species (ROS) is central to cerebral ischaemia-induced oxidative stress in the brain 10–13. It has been demonstrated that oxidative modification in conserved cysteine residues potentially induces nuclear export of class II HDACs and subsequent regulation of transcription factors 14. Doyle *et al*. have also reported that oxidative stress can inactivate class I HDAC1, 2 and 3 and thus antagonize their transcriptional repressor function 15. However, the role of NADPH oxidase on the regulation of HDACs in ischaemic stroke keeps unknown. Therefore, in this study, we used an *in vivo* middle cerebral artery occlusion (MCAO) model and *in vitro* cell cultures by oxygen glucose deprivation (OGD) to investigate the expression patterns of individual HDAC in ischaemic brains and further provide evidence that NADPH oxidase-mediated down-regulation of HDAC4 and HDAC5 expression contributes to cerebral ischaemia/reperfusion (I/R) injury *via* high-mobility group box 1 (HMGB1) signalling pathway.

## Materials and methods

### Animal models for transient focal cerebral ischaemia

Transient middle cerebral artery occlusion (MCAO) was induced in male Sprague–Dawley rats (250–280 g) provided by Laboratory Animals Center of Shandong University as described previously 16. All procedures were pre-approved by the Institutional Animal Use Committee. A successful occlusion was indicated by a decrease in the regional cerebral blood flow (rCBF) to <20% of the baseline by transcranial laser-Doppler (Perimed, Jarfalla, Sweden) measurement in the area of cerebral cortex supplied by the MCA. After 2 hrs of MCAO, the suture was carefully removed to restore blood flow. Reperfusion was confirmed by an immediate increase in rCBF. During and after the surgery, rectal temperature was controlled with a homeothermic blanket and kept at 37°C until the complete recovery of the animal from the anaesthesia. After reperfusion, the rats were anaesthetized and then decapitated. For apocynin treatment, 3 mg/kg bodyweight (Sigma-Aldrich, St Louis, MO, USA) was administered by intraperitoneal injection 30 min. before suture withdrawal. The concentration of apocynin was chosen based on our preliminary experiments and previous studies 17.

### Infract volume and neurological function assessment and HE staining

Stroke outcome was assessed at 24 hrs after reperfusion using cerebral infarct volume and a 4-tiered neurological scoring system and HE staining for cerebral injury assessment as described previously 18.

### Cell culture and treatments

The neuron-like rat pheochromocytoma cell line PC12 was cultured at 37°C in DMEM (Invitrogen, Gaithersburg, MD, USA) supplemented with 10% foetal bovine serum (Invitrogen), 100 U/ml penicillin and 0.1 mg/ml streptomycin (Sigma-Aldrich). During differentiation, cells were cultured in a differentiation medium (DMEM supplemented with 1% serum) containing nerve growth factor (NGF; 50 ng/ml) 19. Differentiated PC12 cells were then transfected with pCMV6-HDAC4 or pCMV6-HDAC5 plasmid (OriGene Technologies, Rockville, MD, USA) by Lipofectamine 2000 (Invitrogen) for 24 hrs. Different cell treatment conditions were used in this study: (i) treated with HMGB1 (2 μg/ml for 24 hrs); (ii) pre-treated with apocynin (100 μM) for 30 min. and then cultured by the model of oxygen glucose deprivation (OGD) as described previously 17.

### RNA extraction and real-time RT-PCR

Total RNA was isolated from cells and tissues (ischaemic core and penumbra or normal controls) using TRIzol reagent (Invitrogen) and then reverse transcribed with a cDNA synthesis kit (Bio-Rad, Hercules, CA, USA) as described previously 20. The mRNA levels for target genes were analysed by real-time quantitative RT-PCR using a Bio-Rad iCycler system (Bio-Rad) according to the manufacturer's manual. Negative controls without adding cDNA or primers were performed to verify the specificity of amplification. Level of the housekeeping gene GAPDH was used as an internal control for the normalization of RNA quantity and quality differences among the samples. Equal amplification efficiencies of GAPDH and target genes were tested, and optimization of primers and cDNA concentrations was performed in preliminary experiments. Data were analysed by the same real-time PCR detection system. The cycle threshold (Ct) values were used for calculation of gene expression in accordance with the ΔΔCt method as described previously 21. The specific primers for target genes in this study are listed in Table 1.

**Table 1:** Primer pairs of rat HDAC family used for real time RT-PCR in this study

Genes	Genbank Accession No.	Forward	Reverse
HDAC1	NM_001025409.1	GCGAGCAAGATGGCGCAGACT	GTGAGGCTTCATTGGGTGCCCT
HDAC2	NM_053447.1	CTCCGGGCTGTCCTTGCTGC	GCCGCCTCCTTGACTGTACGC
HDAC3	NM_053448.1	ACCAGGCCTCCCAGCATGACA	CCGGGAAACACAGGGCAGTCG
HDAC4	NM_053449.1	CACCGTGCCCAGCACTCCAG	GGCCTGTGACAAGGGGCGTC
HDAC5	NM_053450.1	TTCTTCAACTCCGTAGCC	TCCCATTGTCGTAGCG
HDAC6	XM_228753.6	TGTGGCTGCCCGCTATGCAC	GGGGCCAGAACCGACCATGC
HDAC7	XM_003750405.1	ACCCAACCTCAATGCC	GATGCCAACGGAAAGG
HDAC8	NM_001126373.2	CCAGCCACAGAAGGGATA	TTCCGTCGCAATCGTAAT
HDAC9	NM_001200045.1	GTCCCTGCCCAATATCAC	GCTGTTCGGTTTGCCCTC
HDAC10	NM_001035000.1	CCGGCAGAGGGCGTGTTGAG	CAAGGCAGCTGTCAGGCGCT
HDAC11	NM_001106610.2	ACAACCGCCACATCTAC	AGGGACCTCCTCACATT
GAPDH	NM_017008.4	TGCATCCTGCACCACCAACTGC	ACAGCCTTGGCAGCACCAGTGG

### Western blot analysis

Western blotting was performed as described previously 22. Primary antibodies to – HDAC1, HDAC2, HDAC8, HDAC9, HDAC11, HMGB1 and NOX2 (1:1000 dilution; abcam, Cambridge, MA, USA), HDAC3, HDAC4, HDAC5, HDAC6, HDAC7, and HDAC10 (1:1000 dilution, ProteinTech Group, Chicago, IL, USA), and secondary antibodies horseradish peroxidase-labelled antimouse IgG or antirabbit IgG (1:6000 dilution, ProteinTech Group) were used in this study. To document the loading controls, the membrane was reprobed with a primary antibody against housekeeping protein β-actin.

### Immunohistochemistry and immunofluorescence staining

Primary polyclonal antibody HDAC4 or HDAC5 (1:150 dilution) was used in this study. Staining was performed on tissue sections as described 17. To determine the lineage of HDAC4/5-positive cells, additional staining of neurons was performed using anti-NeuN (Invitrogen, Carlsbad, CA, USA), astrocyte was performed by anti-GFAP, whereas anti-CD11b was used to stain microglia.

### Measurements of NADPH oxidase activity

NADPH oxidase activity was determined by measurement of superoxide (O_2_^−^) production in brain or cell homogenates. Fluorescence spectrometry for O_2_^−^ production was performed by using a modified DHE fluorescent spectrometric assay as described 23.

### Enzyme-linked immunosorbent assay of HMGB1

High-mobility group box 1 release was determined in cell culture supernatant by a specific anti-HMGB1 enzyme-linked immunosorbent assay (ELISA; IBL International, Germany) according to the manufacturer's protocol 24. Cerebrospinal fluid (CBF) samples and serum samples were obtained as described previously 25, 26 and HMGB1 levels in samples were measured using ELISA kit.

### Flow cytometry

Cell death was determined by propidium iodide (PI)-Annexin V staining as described previously 24.

### Statistics

Data are expressed as means ± SE. The significance of the differences in mean values between and within multiple groups was examined by one-way anova followed by Duncan's multiple range test. *P* < 0.05 was considered statistically significant.

## Results

### Expression patterns of HDACs in ischaemic brain of rats subjected to focal cerebral ischaemia reperfusion

**Fig. 1:**
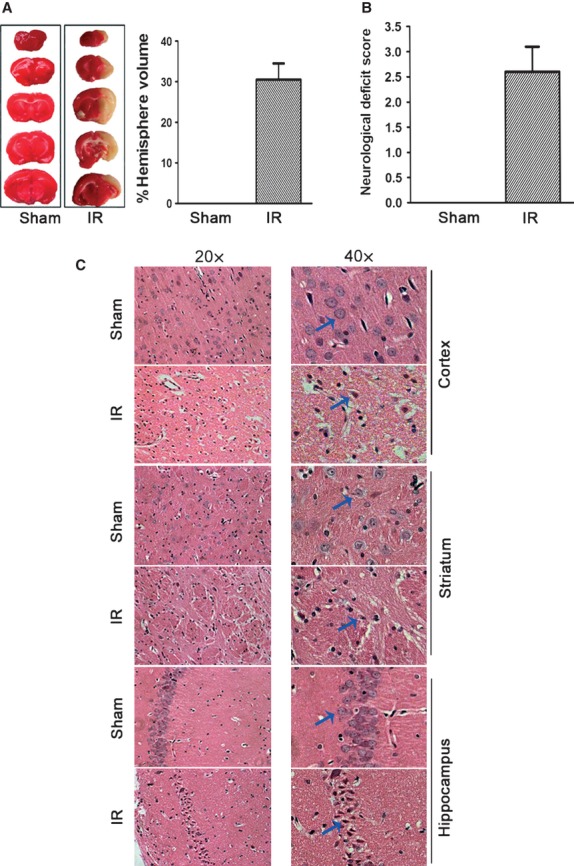
Characterization of brain injuries after focal cerebral ischaemia reperfusion by MCAO. (**A**) Representative photographs of TTC staining (left panel) and calculated infarct volume showing increased cerebral infarct volume at 24 hrs of reperfusion after MCAO in male Sprague–Dawley rats. (**B**) Neurological deficit scores in rats after cerebral ischaemia reperfusion. (**C**) HE staining showed morphological features of injured neurons after ischaemia/reperfusion in different specific brain regions including prefrontal cortex, striatum and hippocampus.

**Fig. 2:**
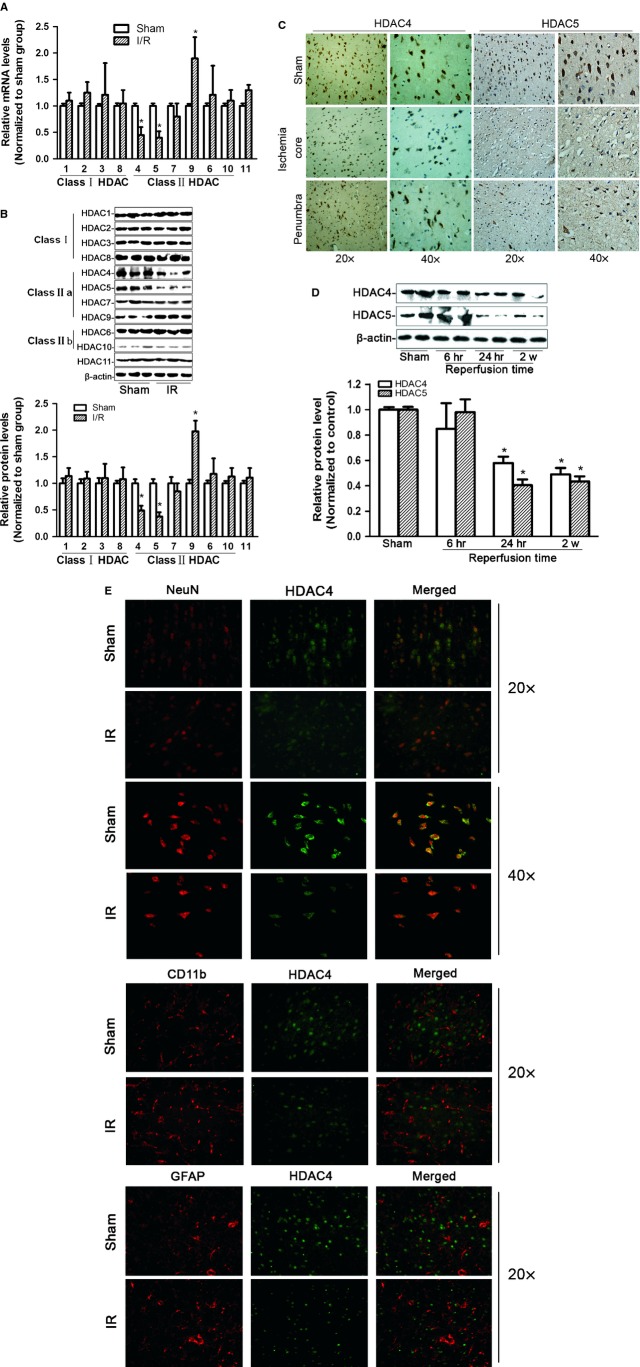
Expression patterns of HDACs in ischaemic brain of rats subjected to focal cerebral ischaemia reperfusion. (**A**) Real-time RT-PCR analysis of HDAC1-11 mRNA levels from total RNAs extracted from ischaemic core and penumbra in the brain, among Zn^2+^-dependent HDACs (HDAC1-11), mRNA levels of HDAC4 and HDAC5 were significantly decreased; HDAC9 expression was remarkably increased. (**B**) Representative Western blot gel documents and summarized data showing the protein levels of HDAC1-11 in the ischaemic core and penumbra of the ischaemic brain. (**C**) Immunohistochemistry for HDAC4 and HDAC5 at 24 hrs of reperfusion after MCAO in ischaemic core and penumbra in the brain. (**D**) Representative Western blot gel documents and summarized data showing the protein levels of HDAC4 and HDAC5 in the ischaemic core and penumbra of the brain at different reperfusion time-points. **(E**) Immunofluorescent staining showing cellular localization of HDAC4 in the cortex from ischaemic brain and normal subjects, indicating that HDAC4 was expressed in neurons rather than astrocytes and microglia. *P < 0.05 *versus* sham-operated rats (*n* = 8).

As shown in Figure 1A, infarction volume at 24 hrs after reperfusion was 35.6 ± 4.1%. Neurological deficit score also confirmed the neurological dysfunction after MCAO (Fig. 1B). HE staining further showed morphological features of injured neurons after I/R in different specific brain regions including prefrontal cortex, striatum and hippocampus, all of which are predominantly injured in focal ischaemia. Arrowheads indicated the normal cells in sham-operated group and injured cells in I/R group. Significant cellular body shrinkage and condensation of dark nuclei were observed after I/R. To determine the expression patterns of HDAC family in ischaemic brain (ischaemic core and penumbra), real-time RT-PCR and Western blot analyses were performed. As shown in Figure 2A and B, among Zn^2+^-dependent HDACs, the expression of HDAC4 and HDAC5 was significantly decreased. The levels of HDAC1, 2, 3, 6, 7, 8, 10 and 11 had no significant difference at this experimental condition. Our immunohistochemical studies further confirmed the decreased levels of HDAC4 and HDAC5 in ischaemic core and penumbra of the ischaemic hemisphere (Fig. 2C). We then assessed the expression levels of HDAC4 and HDAC5 at different time-points after reperfusion, our results indicated that HDAC4 and HDAC5 expression still kept low expression at 2 weeks after reperfusion as shown in Figure 2D. To identify cell types in which HDAC4 and HDAC5 were expressed, dual immunofluorescent staining was performed. We identified neurons by staining for neuronal nuclear marker NeuN, astrocytes by GFAP and activated microglia/macrophages by CD11b. It was found that HDAC4 (Fig. 2E) and HDAC5 (data not shown) were predominantly expressed in neurons. No colocalization of HDAC4 or HDAC5 was observed in astrocytes or microglia. Collectively, these results suggest that neuron is a major resource of HDAC4 or HDAC5 in the brain. In addition, compared with sham group, we found that both HDAC4 and HDAC5 levels were significantly decreased in neurons of ischaemic brain.

### Regulation of HDAC4/HDAC5 and HMGB1 expression is associated with NADPH oxidase activity in differentiated PC12 cells by OGD

**Fig. 3:**
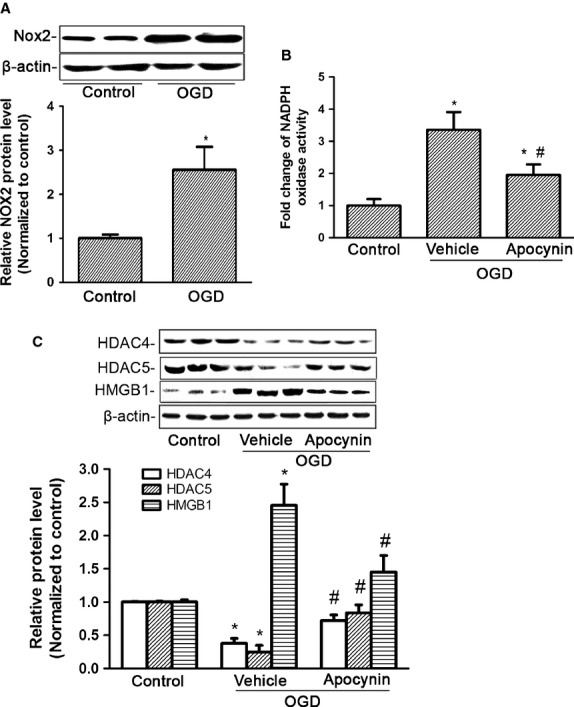
Regulation of HDAC4/HDAC5 and HMGB1 expressions is associated with NADPH oxidase activity in PC12 cells by OGD. (**A**) Representative Western blot gel documents and summarized data showing NOX2 protein levels in PC12 cells cultured by the model of OGD. (**B**) Summarized data showing the effect of apocynin on NADPH oxidase activity in PC12 cells. (**C**) Representative Western blot gel documents and summarized data showing the expression levels of HDAC4, HDAC5 and HMGB1 in PC12 cells. *P < 0.05 *versus* control, #*P* < 0.05 *versus* cells cultured by the model of OGD (*n* = 6).

To examine whether HDAC4 and HDAC5 are redox-sensitive proteins and regulated by NADPH oxidase activity, NADPH oxidase inhibitor apocynin was used in this study. As shown in Figure 3A and 3B, NOX2 (gp91^*phox*^) expression was markedly enhanced when differentiated PC12 cells were cultured by the model of OGD (2 hrs OGD/12 hrs reoxygenation) and apocynin inhibited OGD-induced NADPH oxidase activity. We further found that OGD significantly decreased HDAC4 and HDAC5 expression, which can be recovered by apocynin (Fig. 3C). Moreover, we assessed whether HMGB1, a central and necessary mediator of tissue damage following acute injury, was associated with NADPH oxidase activity, our results showed that apocynin attenuated OGD-induced HMGB1 expression (Fig. 3C).

### HDAC4 and HDAC5 protect cells from death through reducing HMGB1 expression and release

**Fig. 4:**
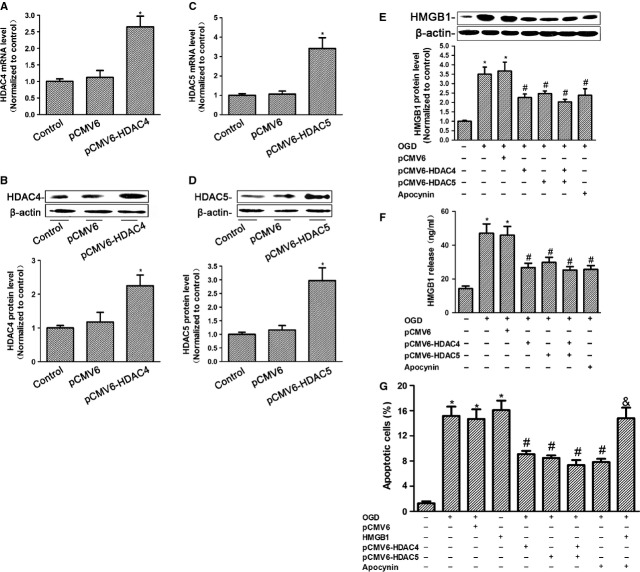
The effects of NADPH oxidase-mediated HDAC4/5 signalling on the expression and release of HMGB1. (**A**) Quantitative RT-PCR analysis of HDAC4 mRNA level in pCMV6-HDAC4 transfected PC12 cells. (**B**) Representative Western blot gel documents and summarized data showing the relative HDAC4 expression in pCMV6-HDAC4 transfected PC12 cells. (**C**) Quantitative RT-PCR analysis of HDAC5 mRNA level in pCMV6-HDAC5 transfected PC12 cells. (**D**) Representative Western blot gel documents and summarized data showing the relative HDAC5 expression in pCMV6-HDAC5 transfected PC12 cells. (**E**) Representative Western blot gel documents and summarized data showing the effect of NADPH oxidase inhibitor apocynin, HDAC4 and HDAC5 on the expression of HMGB1 in PC12 cells cultured by the model of OGD. (**F**) Summarized data showing the release of HMGB1 in the cell culture supernatant measured by ELISA. (**G**) Summarized data showing cell apoptosis determined by flow cytometric analysis indicating that HDAC4 and HDAC5 protect cell from death in PC12 cells by OGD. *P < 0.05 *versus* control, #*P* < 0.05 *versus* cells cultured by the model of OGD, &*P* < 0.05 *versus* cells cultured by the model of OGD and apocynin treatment (*n* = 6).

To further investigate the role of HDAC4 and HDAC5 on cell fates, forced expression of HDAC4 and HDAC5 was used in this study. By real-time RT-PCR and Western blot analyses, HDAC4 and HDAC5 mRNA and protein levels were significantly increased in pCMV6-HDAC4 or pCMV-6 HDAC5 transfected PC12 cells, respectively (Fig. 4A–D). Then, we detected the effect of HDAC4 and HDAC5 on HMGB1 expression and release. It was found that overexpression of HDAC4 or HDAC5 significantly attenuated OGD-enhanced HMGB1 expression (Fig. 4E) and release (Fig. 4F) in PC12 cells. Inhibition of NADPH oxidase activity showed the similar effects. By flow cytometric analyses, both cell cultured under OGD condition or treated by HMGB1 could induce cell apoptosis. OGD-induced apoptosis was markedly ameliorated by overexpression of HDAC4 or HDAC5. Moreover, we found that HMGB1 reversed apocynin-reduced apoptosis under OGD condition, indicating that NADPH oxidase-reduced HDAC4 and HDAC5 expression, at least in part, contributes to cell death *via* HMGB1 signalling pathway (Fig. 4G).

### Inhibition of NADPH oxidase activity ameliorated cerebral ischaemia/reperfusion injury *via* regulation of HDAC4/5 and HMGB1

**Fig. 5:**
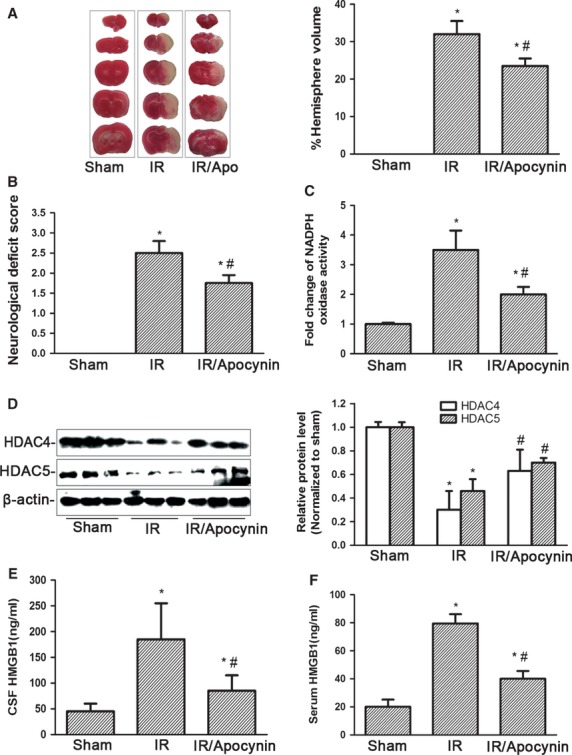
Inhibition of NADPH oxidase activity ameliorates cerebral ischaemia/reperfusion injury and decreased HDAC4/HDAC5 and HMGB1 release. (**A**) Representative photographs of TTC staining and calculated infarct volume in rats with apocynin treatment after cerebral ischaemia/reperfusion. (**B**) Neurological deficit scores in rats with apocynin treatment after cerebral ischaemia/reperfusion. (**C**) Summarized data showing the effect of apocynin on NADPH oxidase activity in core and penumbra of the brain. (**D**) Representative Western blot gel documents and summarized data showing the protein levels of HDAC4 and HDAC5 in core and penumbra of the brain. (**E**) The HMGB1 levels in cerebrospinal fluid were determined using ELISA. (**F**) Serum levels of HMGB1 at 24 hrs after reperfusion were determined using ELISA. *P < 0.05 *versus* sham-operated rats, #*P* < 0.05 *versus* ischaemic rats (*n* = 8).

In this study, we treated rats with apocynin at a dose of 3.0 mg/kg 30 min. before reperfusion as reported 27. Firstly, relevant physiological parameters among all groups were assessed. It was found that physiological variables were not significantly different between vehicle and apocynin-treated rats before MCAO, during MCAO or 24 hrs after reperfusion (data not shown). However, apocynin significantly reduced infarct volume (Fig. 5A) and neurological deficit score (Fig. 5B). Cerebral ischaemia-induced NADPH oxidase activity was markedly attenuated by apocynin (Fig. 5C). We further found that apocynin significantly recovered HDAC4 and HDAC5 levels (Fig. 5D). Together with our preliminary studies showing that apocynin had no effect on HDAC4 and HDAC5 expressions in rats under the sham-operated condition, our results suggest that NADPH oxidase activity is involved in the regulation of HDAC4 and HDAC5 expression after cerebral ischaemia reperfusion. Finally, determination of HMGB1 using ELISA assay showed that HMGB1 levels were significantly increased in the cerebrospinal fluid at 24 hrs after reperfusion. Treatment with apocynin decreased the HMGB1 levels in cerebrospinal fluid (Fig. 5E). The increase in serum HMGB1 levels after ischaemia was also observed, but the serum levels of HMGB1 were suppressed by apocynin treatment (Fig. 5F).

## Discussion

Histone deacetylases inhibitors have displayed neuroregenerative and neuroprotective properties in animal models for various neurological diseases 28–30. By contrast, there is accumulating evidence that HDAC enzymes exert protective effects in several pathological conditions including ischaemic stress 31, 32. Mice expressing an HDAC1 gain-of-function transgene exhibit potent protection against DNA damage and neurotoxicity in cultured neurons and in an *in vivo* model of ischaemia 33. The mixed results indicate the specific roles of each HDAC protein and the possible function of distinct histone modification in different diseased states. However, up to date, reports about the subtype of HDACs associated with ischaemic stroke are limited. Therefore, in this study, we investigated the expression patterns of HDACs in ischaemic brain and our results showed that among Zn^2+^-dependent HDACs, HDAC4 and HDAC5 were significantly decreased both *in vivo* and *in vitro*, which can be reversed by NADPH oxidase inhibitor, apocynin. We further investigated the roles of HDAC4 and HDAC5 on the regulation of HMGB1, a central and necessary mediator of tissue damage following acute, sterile injury 34, showing that both HDAC4 and HDAC5 increased the viability of cells through inhibition of HMGB1 expression and release. This study for the first time provides evidence that HDAC4 and HDAC5 serve as central target molecules that link NADPH oxidase-derived ROS to HMGB1 signalling pathway.

Class IIa HDACs possess both N-terminal domains that interact with transcription factors and C-terminal nuclear export signals enabling shuttle between the nucleus and cytoplasm in a phosphorylation-dependent manner 5. Class IIa HDACs act as transcriptional activators or repressors, but in either situation, these enzymes primarily control gene expression by recruiting other proteins as corepressors or coactivators. HDAC4 and HDAC5 belong to class IIa HDACs. HDAC4 has been reported to protect neurons from cell death by inhibiting cyclin-dependent kinase 1 (CDK1) and cell-cycle progression 35 and regulate the survival of retinal neurons in the mouse in normal and pathological conditions partly by increasing HIF-1α stabilization 36. HDAC5 is considered as a critical regulator of adaptive responses to chronic stress and cocaine consumption 37, 38. Therefore, HDAC4 and HDAC5 appear to play important roles in the peripheral and CNS. In this study, our results show that HDAC4 and HDAC5 are significantly decreased in both *in vivo* and *in vitro* studies. Moreover, we find that HDAC4 and HDAC5 protect cells from death under OGD condition. However, the molecular mechanisms to decrease HDAC4 and HDAC5 expression in ischaemic brain require further investigation.

It is well known that NADPH oxidase-produced ROS contributes to cellular signalling, affecting cellular functions including gene expression in different pathophysiological conditions including ischaemic stroke 39. Given the role of ROS on the regulation of HDAC activity, we examine whether HDAC4 and HDAC5 are redox-sensitive proteins and are potential molecular targets of NADPH oxidase-derived ROS for the regulation of cellular events. In this study, we find that NADPH oxidase activity is dramatically increased after cerebral I/R. Moreover, we demonstrate that the regulation of HDAC4 and HDAC5 expression is associated with NADPH oxidase activity, indicating the contributing role of NADPH oxidase on the regulation of HDAC4 and HDAC5 in cerebral I/R rats. These results suggest that neuroprotective effects of inhibition of NADPH oxidase activity after ischaemia reperfusion may go through, at least in part, the regulation of HDAC4-/HDAC5-mediated histone acetylation and gene transcription.

High-mobility group box 1, originally described as a nuclear protein that binds to and modifies DNA, is now regarded as a vital mediator of inflammation by acting as a cytokine in clinical conditions such as autoimmunity, cardiovascular disease and cancer 40–42. Recent studies have also indicated that HMGB1 is involved in the pathogenesis of ischaemic stroke 43. HMGB1 is expressed and released in various types of cells with autocrine/paracrine networks involving other factors. HMGB1 is expressed in the nucleus in normal conditions and rapidly translocates to the cytoplasm and release (or secrete) from dying cells into extracellular milieu in the setting of ischaemic stroke. The extracellular HMGB1 may act on the neighbouring neurons, causing aggravated cerebral dysfunction through different receptor signalling pathways 44. Moreover, the release of HMGB1 is also observed from neurons under OGD conditions 45. Down-regulation of HMGB1 mediated by shRNA or monoclonal antibody causes diminished infarct size, reduced neuroinflammation, indicating that HMGB1 has a critical role in ischaemia 46. Nevertheless, the mechanisms governing HMGB1 release in cerebral I/R are not clear. Several studies have showed that HMGB1 is actively transported between the nucleus and cytoplasm following detachment from loosened chromosomes by histone acetylation 47, 48. Very recently Evankovich *et al*. have reported that decreased HDAC activities in hepatocytes following liver I/R contribute to the hyperacetylation and subsequent release of HMGB1 31. Therefore, it is possible that this signalling pathway is also involved in cerebral I/R injury. In this study, we manipulate HDAC4 and HDAC5 levels by overexpression and address the role of HDAC4 and HDAC5 on the regulation of HMGB1. Our results indicate that forced expression of HDAC4 and HDAC5 decreases not only HMGB1 release but also expression, indicating the contributing role of HDAC4 and HDAC5 on the regulation of HMGB1 function in ischaemic stroke.

There is an increasing number of evidence showing that besides necrosis, apoptosis does significantly contribute to the cell death subsequent to I/R injury 49. Although focal and global cerebral ischaemia stroke ultimately results in dysfunction or loss of brain cells, there are significant differences in the mode of cell death. Global cerebral ischaemia causes delayed neuronal death selectively in CA1 hippocampal neurons. Although DND exhibits distinct morphological features from apoptosis, the molecular mechanisms involved in the apoptotic process may contribute to delayed neuronal death 50. On the other hand, most of the cells in the ischaemic core undergo necrosis in focal cerebral ischaemia. Meanwhile, cell death in the penumbra largely depends on the activation of cell death programmes leading to apoptosis. Evidence has been shown that activation of apoptotic pathways occurs in the penumbra 51. The abortion of apoptotic process in the ischaemic core is possibly caused by the severe impairment of energy levels that may cause a shift towards secondary necrosis from apoptosis 49, 52. In this study, we found that HDAC4 and HDAC5 were decreased both in ischaemia core and penumbra. Considering that HMGB1 has been demonstrated to induce apoptosis in PC12 cells 53, we further detect the effect of HDAC4/5 on PC12 cell apoptosis. Our results indicated that NADPH oxidase-reduced HDAC4 and HDAC5 expression, at least in part, contributes to cell apoptosis *via* HMGB1 signalling pathway.

In addition, we observe that HDAC9, another member in class IIa HDACs, is significantly increased under cerebral I/R. Normally, HDAC9 transcripts are expressed at high levels in the brain and skeletal muscle 54 and associated with poor survival 55, but very little is known about its actions in the brain. The histone deacetylase-related protein, an alternatively spliced and truncated form of HDAC9 that lacks a C-terminal catalytic domain, is protective against apoptosis in cultured neurons 56. Of note, very recent genome-wide association study identifies a variant in HDAC9 associated with large-vessel ischaemic stroke 57, 58. However, the molecular pathways that are affected by the identified genetic variants are not yet pinpointed, and the role of HDAC9 is ischaemic stroke keeps unknown. Therefore, it is very important to make further study of this protein in the brain. At present, our research group is elucidating the role and molecular mechanisms of HDAC9 in ischaemic stroke.

In summary, this study for the first time demonstrates that NADPH oxidase-mediated HDAC4/5 expression and activity contribute to cerebral ischaemia injury *via* HMGB1 signalling pathway, suggesting a new pathogenic pathway governing cellular responses to ischaemic stress, which may provide an important therapeutic strategy to prevent tissue damage in ischaemic stroke. In particular, this study tells us for the future research of HDAC therapeutics that it is important to elucidate the role of individual HDACs within the brain, and the development of HDAC inhibitors with improved specificity is required to develop effective therapeutic strategies to treat brain disease.
